# Assessment of the Mechanical Properties and Durability of Cement Mortars Modified with Polyurethane Foam Waste

**DOI:** 10.3390/ma19030491

**Published:** 2026-01-26

**Authors:** Gabriela Rutkowska, Barbara Francke, Filip Chyliński, Mariusz Żółtowski, Hanna Michalak, Agnieszka Starzyk, Michał Musiał, Oskar Sierakowski

**Affiliations:** 1 Institute of Civil Engineering, Warsaw University of Life Sciences, Nowoursynowska 166, 02-787 Warsaw, Poland; barbara_francke@sggw.edu.pl (B.F.); agnieszka_starzyk@sggw.edu.pl (A.S.);; 2 Building Research Institute, Filtrowa 1, 00-611 Warsaw, Poland; f.chylinski@itb.pl; 3 Faculty of Architecture, Warsaw University of Technology, Plac Politechniki 1, 00-611 Warsaw, Poland; hanna.michalak@pw.edu.pl; 4 KERAKOLL POLSKA Sp. z o.o., ul. Katowicka 128, 95-030 Rzgów, Poland; michal.musial@kerakoll.pl

**Keywords:** polyurethane foam waste, waste utilisation cement mortar, durability, mechanical performance, microstructure analysis

## Abstract

In the era of growing demand for sustainable solutions in construction, increasing attention is being paid to the potential use of waste materials as components of building composites. This article presents the results of a study on the impact of ground polyurethane foam waste on the mechanical properties and durability of cement mortars. The waste, derived from industrial production processes, was used as a partial replacement for fine aggregates in various proportions. The analysis included bulk density, compressive and flexural strengths, water absorption, and resistance to freeze–thaw cycles. The results indicate that adding waste reduces the density of the mortar, which can be advantageous in applications requiring lightweight materials. The most favourable balance of strength retention, density reduction, and frost resistance was observed with a 1% addition, as the mortar maintained good mechanical performance and freeze–thaw durability while achieving reduced weight. Higher waste content (2–3%) led to significant deterioration of the mechanical properties due to increased porosity. All samples exhibited increased strength after 25 freeze–thaw cycles, possibly due to continued hydration under moist low-temperature conditions. The analysis of the microstructure of cement coatings with the addition of polyurethane foam enabled the explanation of the causes of the observed changes in physico-mechanical properties resulting from ageing factors. This study suggests that small amounts of waste can be effectively used to produce lightweight and environmentally friendly construction materials, supporting circular economy practices.

## 1. Introduction

Due to increasing pressure to reduce resource consumption and the environmental impact of building materials, developing solutions with lower energy demand and a reduced carbon footprint is gaining importance. Concrete and cement mortars are widely used in the construction industry; their production is associated with high CO_2_ emissions and significant consumption of non-renewable raw materials, such as mineral aggregates and sand. One of the key challenges is to reduce the carbon dioxide emissions associated with the production of cement, the basic component of concrete and mortar. Currently, global Portland cement production is approximately 4.6 billion tons per year, and its manufacturing process is responsible for approximately 8% of total global CO_2_ emissions. It is estimated that each ton of cement generates between 0.6 and 0.8 tons of this greenhouse gas, which places the cement industry among the top carbon-intensive industries worldwide [1].

Therefore, one of the key directions for the development of construction technologies is the search for alternative components derived from industrial and consumer waste that can replace traditional components while maintaining or improving product performance. Technological and economic development have contributed to an improvement in the quality of life, but at the same time, they have generated numerous environmental problems, including an increase in waste. Although industrial waste has often been landfilled in the past, this is no longer universally the case. Many countries now actively promote waste recovery, and the construction sector is a leading example of successful reuse of industrial by-products. This shift supports the transition from a linear to a circular economy, defined by the European Parliament as a system based on reusing, repairing, refurbishing, and recycling materials for as long as possible. The EU Framework Directive 2008/98/EC promotes the waste hierarchy, prioritising recycling, reuse, and material and energy recovery as effective methods for reducing their environmental impact [2,3,4,5]. Among the problematic waste fractions, polyurethane (PU) foam, which accounts for a significant portion of the plastics market, is particularly noteworthy. According to data from the European Polyurethane Manufacturers’ Association, approximately 67% of total polyurethane production is in the form of foams, which are widely used in building insulation (as ready-to-use boards and spray foam [6,7,8,9]), as building adhesives [10], or in furniture, and automotive applications [11,12]. They are classified as flexible (open-cell) or rigid (closed-cell) [13,14,15,16]. These foams are difficult to process due to their low density and large volume, making it impossible to melt and recycle them using traditional mechanical methods. Consequently, most PU waste ends up in landfills or is incinerated, resulting in the emission of harmful substances and the waste of material potential [17,18,19]. Polyurethane waste (PUW) generated in the automotive, construction, packaging, and furniture industries is a particular challenge.

Owing to its durability, complex chemistry, and low biodegradability, polyurethane is difficult to dispose of. The methods used so far for the management of PUW include combustion with energy recovery and processing into secondary foams with the use of chemical binders such as PAPI (polymethylene polyphenylisocyanate that contains MDI) [20].

In recent years, there has been growing interest in the reuse of shredded PU foam as an additive to cement mortars. Several experimental studies have been reported in the literature, demonstrating that the incorporation of foam fractions, typically in the form of particles with a size range of 1–6 mm, can significantly impact the physical and mechanical properties of mortars. In particular, the following has been observed:

- The use of PU waste leads to a reduction in the volume density of the mortar, which makes it potentially useful in lightweight structures and prefabricated insulation elements.
- The thermal insulation of the material is improved, which can be used in energy-efficient construction,
- Simultaneously, the mechanical parameters deteriorate, particularly the compressive and bending strengths, depending on the amount and type of additive used [18,19,20,21].

Due to their low density, porosity, and excellent thermal insulation properties, waste polyurethane (PU) foams are a promising component for lightweight cement and gypsum mixes. They can partially replace aggregates, improving insulation and reducing the weight of concrete and mortars [22,23,24,25,26,27,28]. Other applications include soil stabilisation, geopolymers, elastomers, and modified gypsum mortars. In line with circular economy and “zero waste” principles, recycling PU foam in building materials supports sustainable construction. It reduces landfill waste, decreases the demand for natural aggregates, lowers material and disposal costs, and enhances energy efficiency due to better thermal insulation and reduced dead weight. While initial foam costs may be higher, savings in labour, transportation, and energy make PU-modified mortars a cost-effective, environmentally friendly alternative in projects prioritising low weight, insulation, and durability [29,30,31,32,33].

The use of recycling in PU waste management has significant environmental benefits, as it reduces the consumption of primary raw materials and the amount of waste sent to landfills or incinerators [34,35,36,37]. It is estimated that approximately 30% of the global PU foam produced ends up as waste each year. Of this waste, only 33% is recycled, 45% is incinerated, and 22% is disposed of in landfills. However, recycling these materials is challenging because of their complex chemical compositions and the limited efficiency of current processing technologies. Although incineration of PU waste is used on a large scale, it is associated with the emission of harmful substances into the atmosphere, which poses a threat to human health and the environment. Moreover, this process does not always result in the complete decomposition of the material, and ash residues require further disposal [38,39,40].

Polyurethane (PU) foam waste can be recycled physically or chemically. Physical recycling, which does not alter the chemical structure, is simple, low-cost, and eco-friendly [41,42,43,44]. Grinding converts PU foam into particles for fillers or secondary products (e.g., carpet underlays, insulation, packaging), while regrinding produces powder mixed with raw PU to create new foam, particularly for low-density applications. Chemical recycling decomposes polymers via glycolysis, hydrolysis, aminolysis, thermochemical methods, or biodegradation to recover polyol monomers for high-quality PU products [45,46,47]. Using PU foam waste as filler reduces material costs and landfill waste and can improve properties like thermal insulation and vibration damping. Despite these benefits, few studies have explored its use in building materials. Junco and Gadea used PU foam from refrigeration (up to 6 mm) to replace fine aggregates in mortars, showing that it could substitute up to 100% of traditional aggregate after ageing tests [48,49]. Molero et al. [50] found that adding ground PU to plaster decreased density and strength, which may enhance thermal and acoustic insulation.

The aim of this study was to evaluate the potential of polyurethane foam residues generated during the production of bulletproof vests as a functional modifier for cement mortars. In contrast to previous research on PU waste—which has primarily focused on insulation or furniture foams and commonly investigated high replacement levels of fine aggregates—this work examines a previously unstudied type of PU. This unique morphology suggests that such residues may interact with the cement matrix in a manner different from typical PU insulation foams.

A further objective of the study was to investigate the effects of very low PU addition levels (1–3% by aggregate mass). While the existing literature often reports substantial strength reductions at high PU replacement ratios, the potential of minimal dosages to improve selected performance indicators—such as crack resistance, deformability, or freeze–thaw durability—has not been adequately explored. By restricting the PU content to these low levels, the study aimed to determine whether it is possible to enhance the functional behaviour of mortars while maintaining their structural integrity.

The research also sought to provide a broader, more comprehensive durability assessment than is commonly presented in earlier works. For this purpose, the study evaluated the long-term performance of PU-modified mortars under varying temperatures enabling a more holistic understanding of how ballistic foam waste influences the stability of cement-based materials.

Overall, the goal of this study was not only to propose an effective method for utilising a difficult industrial waste stream but also to generate new scientific knowledge regarding the behaviour of a previously unexamined PU waste type at optimised low dosages and its influence on both mechanical properties and durability. By addressing these unexplored aspects, the research advances the current state of the art beyond incremental confirmation of known trends.

## 2. Materials and Methods

### 2.1. Materials

Based on preliminary tests, a reference mortar and three mortars with the addition of polyurethane foam waste were prepared for laboratory testing. The test samples were prepared from the four mortars at 21 °C as follows:

- Previous mixing of both aggregates with approximately half of the water necessary for the mortar was performed for 30 s. The previous mixing of sand with foam was performed to facilitate the homogenization of sand. Finally, cement was added, along with the remaining amount of water. In this case, the duration of mixing was 3 min under laboratory conditions, that is, at a temperature of (21 ± 2) °C and relative humidity of (60 ± 10)%.
- Samples were cured at a temperature of (21 ± 2) °C and relative humidity of (60 ± 10)% after being placed in the moulds. The moulds were covered with foil and left under these conditions until the required curing time was reached, namely 24 h. After that time, all samples were removed from the moulds and placed in containers filled with water for the next 28 d. The following products were used to prepare the test mortars:
  - CEM I 42.5 R cement (manufacturer Cement Ożarów S.A., Ożarów, Poland), meeting the requirements of EN 197-1:2012 [51], with high early strength as given in Table 1 and Table 2 [52].
  - Fine aggregate—river sand (from the Vistula River in Poland) with a grain size of 0–2 mm and a specific density of 2.61 g/cm^3^, determined according to EN 1097-7:2023-04 [53].
  - Drinking water—tap water with properties consistent with the requirements of EN 1008:2004 [54].
  - Polyurethane foam waste with a density of 0.04 g/cm^3^ in the form of non-allergenic shapes, passing through a sieve with 0.063 mm mesh, constituting waste in the production of Kevlar Comfort-style 310 water-repellent polyurethane foam bulletproof vest fillings, with the properties given in Table 3.

The detailed compositions of all tested sets and basic identification data are given in Table 4, and the test sets were designated as follows:

- PUO—without additive—reference mortar;
- PU1—with the addition of polyurethane foam waste in the amount of 1% of the aggregate weight;
- PU2—with the addition of polyurethane foam waste in the amount of 2% of the aggregate weight;
- PU3—with the addition of polyurethane foam waste in the amount of 3% of the aggregate weight.

This interval was chosen based on preliminary laboratory tests and previously published studies indicating that polyurethane additions above approximately 3% lead to a substantial reduction in mechanical performance, making higher contents less relevant for practical mortar applications.

To ensure comparable conditions for all cement mortar series, the total and effective water content in mixtures containing waste polyurethane foam (PU) was measured. PU foam is highly absorbent, which can cause localised water withdrawal from the cement paste, affecting both consistency and cement hydration. To quantitatively assess the material’s sorption capacity, water absorption was measured using the gravimetric method. PU foam samples were dried to a constant mass at (50 ± 2) °C and then immersed in water at (20 ± 2) °C for 5 min, which matches the mortar mixing time. After removal from the water, the surface was gently dried with filter paper and weighed. The sorption capacity (A) was calculated, and the effective water content (Weff) was determined. All measurements were performed in triplicate. The PU foam was added to the mixtures in a partially saturated state to minimise the effect of its porous structure on water withdrawal from the cement paste.

**Figure 1:**
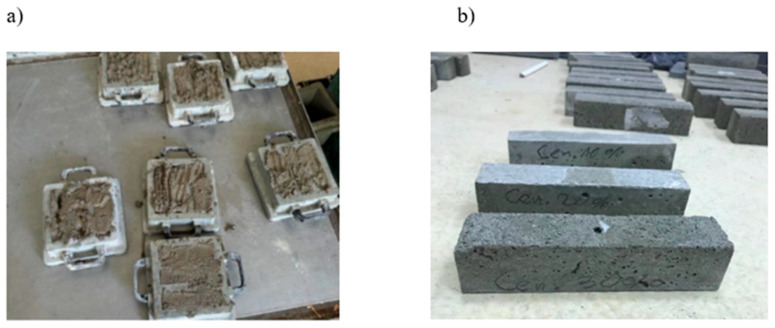
Samples for testing: (**a**) mortar placed in the mould and (**b**) samples of mortar during the maturation period.

To ensure reproducible conditions, water was added to the mixtures containing polyurethane foam waste until an optimal plastic consistency was achieved, as determined by the flow table test according to specify standard (e.g., EN 1015-3:2000 [56]). The prepared mixture was introduced into the moulds (Figure 1a), creating samples with dimensions of 40 × 40 × 160 mm. The samples were left in the moulds for 24 h, after which they were demoulded and placed in water (Figure 1b).

### 2.2. Test Methods

#### 2.2.1. Research Assumptions

The following research assumptions were adopted as part of the analyses presented in this study:

- Comparison of changes in the basic physical properties of mortars, depending on the amount of polyurethane foam addition, in relation to standard mortar;
- Assessment of selected mechanical properties of hardened mortars to determine trends in the preservation of mechanical properties of final products;
- Assessment of resistance to selected ageing factors;
- The analysis of the microstructure of cement coatings with the addition of polyurethane as an essential tool for clarifying the causes of observed changes in physico-mechanical properties resulting from ageing factors.

#### 2.2.2. Tests of Selected Physical Properties of Mortars

The tests of the selected physical properties of the test mortars were treated as the first, preliminary assessment to determine the legitimacy of qualifying for further mechanical and ageing tests for the adopted amount of polyurethane foam waste added to the cement mortar. The obtained values were classified based on a comparison with the values obtained for the reference mortar. The following were evaluated as part of the physical tests:

- The consistency of the cement mortars was tested using the reflow table method, according to EN 1015-3:2000 [56]. It consisted of determining the diameter of the flow of fresh mortar placed on the disc of the spreading table using a special form and subjected to standardised vertical shocks by raising and freely falling the spreading table from a certain height.
- The volume density of fresh mortar, according to EN 1015-6:2000 [57], was determined as the quotient of the mass and volume of the mortar sample after it was placed in a container with standardised dimensions.
- The density of the hardened mortar was determined according to EN 1015-10:2021 [58] as the quotient of the mass of dried hardened mortar and the volume it occupies in the saturated state when immersed in water.
- Water absorption was conducted in accordance with EN 772-21:2011 [59] on specimens with dimensions of 40 mm × 40 mm × 160 mm. For each type of mortar, three samples were selected, removed from the water, and weighed. The samples were then dried to a constant mass and weighed again, after which the water absorbability by weight was determined [59].

#### 2.2.3. Flexural and Compressive Strength Testing

**Figure 2:**
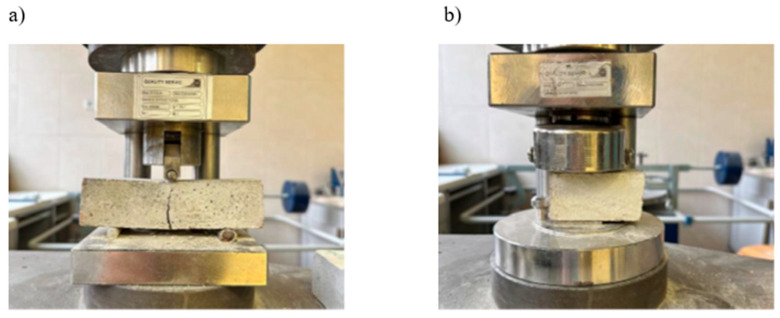
Testing: (**a**) flexural strength and (**b**) compressive strength.

The flexural and compressive strengths were tested according to EN 196-1:2016 [60] to assess the stability of the characteristics of mortars containing polyurethane foam waste. The tests were carried out for four mortars cured at the temperature and relative humidity values of (21 ± 2) °C/(60 ± 10)% after 7, 14, 21, and 28 d of curing, on three samples in each case used for flexural strength tests and six for compressive strength tests. The test was carried out at a speed of (2400 ± 200) N/s on a hydraulic press ZD-40 (produced in GDR) with a maximum load of 10 kN. The specimens used for the flexural and compressive strength tests are shown in Figure 2.

#### 2.2.4. Frost Resistance

**Figure 3:**
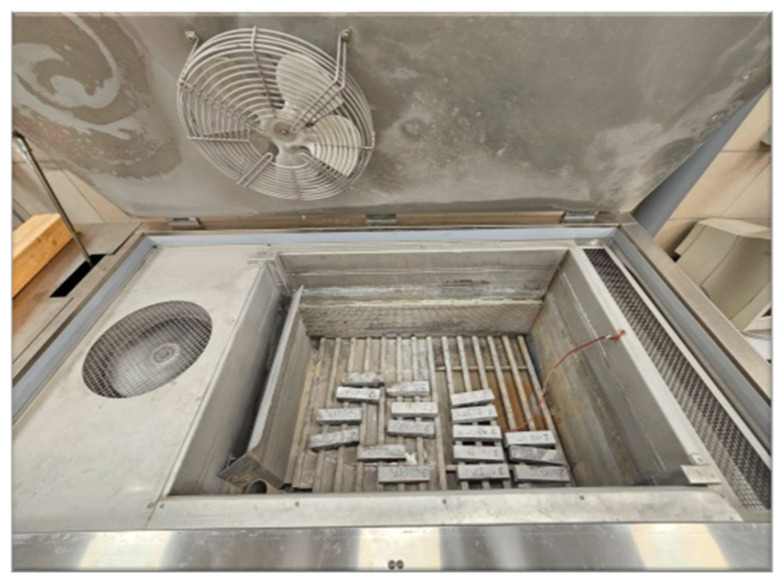
Frost resistance test chamber with samples of tested mortars.

The PN-B/06250 standard [61] was used to assess the frost resistance of the mortars with foam waste. The mortar samples were subjected to cyclic freezing in air and thawing in water for 25 cycles after 28 d of curing. The test was performed in a Toropol K-010 chamber (Poland, Warsaw) (Figure 3). The cycle was controlled using a Jumo Imago 500 microprocessor controller (Poland, Warsaw).

#### 2.2.5. Roughness Analysis

Roughness analysis of the concrete samples was conducted using the Keyence digital imaging microscope of the VHX-6000 series (Osaka, Japan), equipped with a universal zoom lens VH-Z20R/Z20T and the Keyence wide-area 3D measurement system VR-6200 series (Osaka, Japan). The digital imaging microscope enabled the observation and evaluation of existing micro-damages and surface topography, in accordance with the ISO 4287 standard [62]. The specimens for microscopy analysis, measuring 40 mm × 40 mm × 25 mm, were cut from each tested sample.

**Figure 4:**
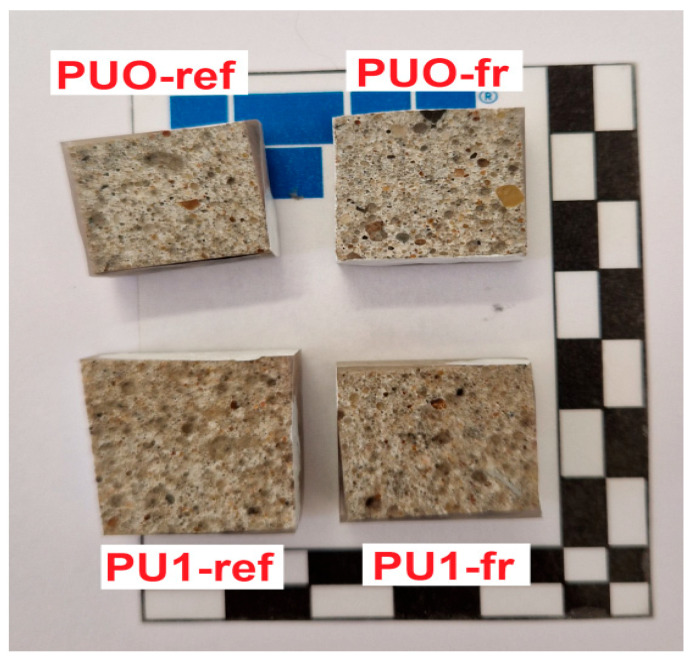
Samples for microscopic examination.

Additionally, from the PUO and PU1 reference samples—as well as PUO-fr and PU1-fr samples after freeze–thaw cycles—smaller pieces with surface dimensions of approximately 20 mm × 20 mm were cut perpendicularly to the troweled surface. These smaller specimens were dried and then placed in resin under vacuum. After the resin had set, the samples were ground and polished to obtain a smooth, uniform surface suitable for microscopic examination. The prepared samples are shown in Figure 4.

#### 2.2.6. Microstructure Examinations

Microscopic examinations were initially conducted using a stereoscopic optical microscope by ZEISS, model Stemi 508 (Oberkochen, Germany). Subsequently, after gold evaporation, the samples were observed with scanning electron microscopy (SEM) by ZEISS, model Sigma 500 VP (Oberkochen, Germany). Backscattered electron (BSE) detector images were acquired. Chemical analysis in micro areas and mapping were performed using an energy-dispersive X-ray (EDX) detector by Oxford Instruments, model Ultim Max 40 (Abingdon, UK).

**Figure 5:**
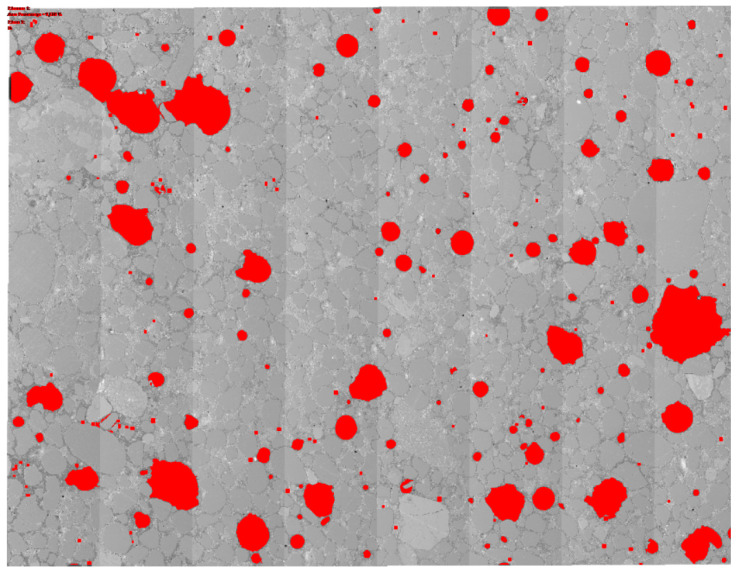
Example of statistical image analysis during processing.

Pore size distribution was assessed using statistical image analysis of stitched SEM images. For this task, ZEN software (blue edition) was used. Total porosity, minimum and maximum, and average and median pore size were determined. For each sample, an area of about 400 mm^2^ was analysed. Figure 5 presents an example of statistical image analysis during processing. The red areas represent areas classified by the programme as empty air spaces, the dimensions of which have been further analysed and processed.

## 3. Results and Discussion

### 3.1. Properties of Fresh Mortar

**Figure 6:**
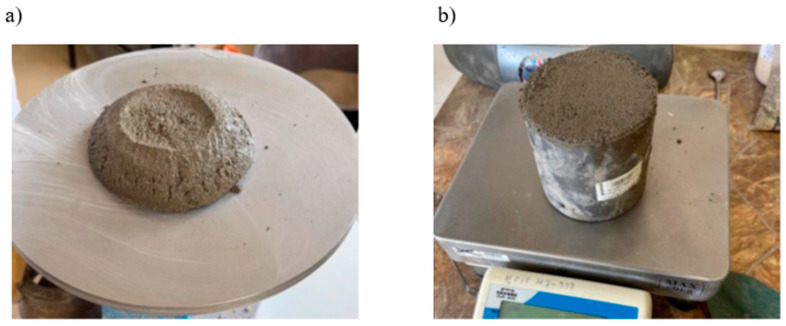
Fresh mortar testing: (**a**) consistency and (**b**) density.

Four series of cement mortars were prepared, with a portion of the mineral aggregate gradually replaced by polyurethane (PU) waste at proportions of 0%, 1%, 2%, and 3% of the aggregate weight (samples: PUO, PU1, PU2, and PU3, respectively). The amount of cement (450 g) and the nominal amount of mixing water (225 g) were kept constant. Because the PU-modified mixtures required additional water to achieve comparable workability, the total water content and the effective water content differed between PUO, PU1, PU2, and PU3. To ensure transparency of the mix design and to allow a meaningful comparison of the tested mortars, both parameters were calculated. The total w/c ratio increased from 0.50 (PUO) to 0.70 (PU3), while the effective w/c ratio—defined as the amount of water available for cement hydration after subtracting the water absorbed by PU waste—ranged from 0.556 to 0.668. These values indicate that part of the added water was not participating in the reaction but was bound physically by the PU particles. The consistency was measured according to the reflow table method, as shown in Figure 6a. The research found that increasing the PU waste content required adding more water (PU1-50 g, PU2-100 g, and PU3-150 g) to keep the mortars’ consistency similar. In the reference PU0 mortar, the measured flow was 12.0 cm, while in the mixtures with polyurethane (PU), it ranged from 11.2 to 11.5 cm. This decrease shows that adding polyurethane filler reduces the mortar’s workability. The flow reduction may be caused by several factors, such as

- The absorbency of the porous waste material;
- The rough and irregular surface of PU particles, which increases internal friction within the mixture;
- The potential aeration of the mixture during mixing.

As the PU content increases, a consistent decrease in the density of the fresh mortar was observed. The PUO reference sample had a density of 2.163 g/cm^3^, as shown in Figure 6b. Introducing only 1% PU (PU1) lowered the density to 2.108 g/cm^3^, while 3% PU (PU3) reduced it further to 1.696 g/cm^3^, which is approximately 22% lower than the reference sample. This decline is due to the lower specific density of PU waste, which replaces the mineral aggregate, and the addition of water needed to maintain workability, which can also increase air content in the mix. It is also possible that the PU material added additional structural porosity to the mortar.

### 3.2. Properties of Mature Mortar

**Figure 7:**
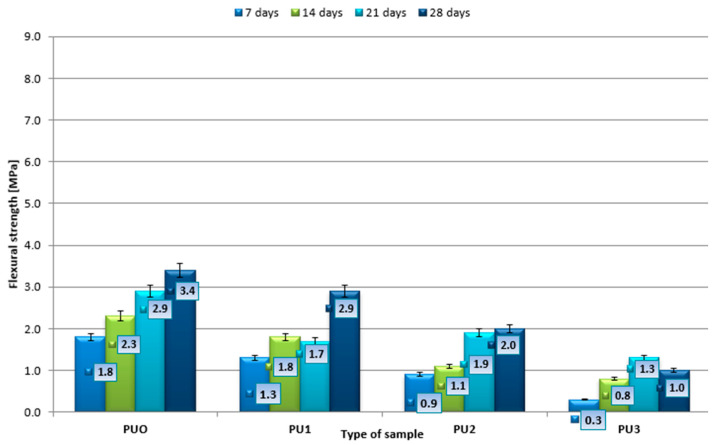
Changes in the average flexural strength during mortar setting.

**Figure 8:**
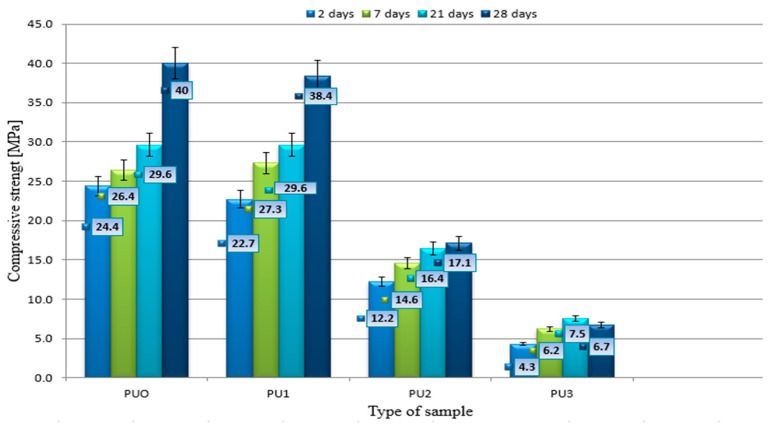
Changes in the average compressive strength during mortar setting.

In the next phase of the study, the impact of PU waste on the strength of mortars at different curing periods (7, 14, 21, and 28 days) was examined. Figure 7 and Figure 8 illustrate the results for flexural and compressive strengths, respectively. The standard deviation from the mean value is marked at the top of each rectangle. To separate the influence of PU replacement from the influence of water demand, the interpretation of the mechanical and durability results in this study was based primarily on the effective w/c ratio, which more accurately reflects the real water available to the cement matrix. Although PU addition required an increase in total mixing water to maintain constant flow, the effective w/c ratios varied only moderately (0.556–0.668), and the reduction in mechanical properties cannot be explained solely by these differences. This confirms that the observed changes in strength and durability are predominantly attributable to the introduction of PU particles and to their interaction with the cementitious matrix (increased porosity, weakened interfacial transition zone, altered microstructure).

Research indicates that the addition of polyurethane foam significantly influences the mechanical properties of cement mortar, particularly its bending strength. The control sample (PUO), which is the mortar without foam addition, displayed the highest strength at each stage of curing. Its strength increased steadily and linearly over time, reaching 3.4 MPa after 28 days. This consistent strength improvement confirms proper cement hydration and the development of the binding matrix, serving as a benchmark for evaluating the effect of incorporating PU foam. In the case of PU1% mortar, containing 1% polyurethane foam, lower strength was observed compared to the control in the early curing stages (1.3 MPa after 7 days). Interestingly, after 21 days, there was a slight decrease in strength, followed by a significant increase to 2.9 MPa after 28 days. This could suggest a delayed maturation process or structural changes within the material. PU2% mortar, containing 2% foam, showed substantially lower strength during the initial period (0.9 MPa after 7 days). However, over time, there was an increase in endurance, reaching 2.0 MPa after 28 d. These findings indicate that a higher foam content slows down matrix densification yet still enables the composite to achieve an acceptable strength after longer curing periods. This suggests that excess foam has a negative impact on the durability and stability of the mortar structure, limiting the usefulness of this mixture in structures subject to bending loads. Adding 3% foam resulted in very low strength from day 7 onwards. Notably, after a slight increase up to day 21, strength decreased to 1.0 MPa after 28 days. This behaviour may be due to excessive porosity, less effective particle packing, and weakened interfacial bonding caused by too much foam. This highlights the adverse effect of over-foaming on the long-term stability and structural integrity of the mortar.

The research allows us to conclude the following:

- A small addition of foam (1%) can be beneficial in non-structural applications or where the lightness or insulation of mortar is important.
- Excess foam (>2%) significantly deteriorates the mechanical properties, limiting the possibility of using such mixtures in construction practices.
- The behaviour of the PU1% sample after 28 d shows that proper formulation tuning can allow for a satisfactory compromise between strength and additional properties (e.g., thermal insulation).

Based on the analysis of results presented in Figure 8 on the compressive strength of cement mortar sample specimens modified with the addition of polyurethane (PU) foam across different maturation periods (2, 7, 21, and 28 d), it is also possible to notice a clear effect of the foam content on the above-mentioned properties. Reference samples (PUOs), which did not contain any additives, showed the highest compressive strength values at all measurement points, reaching 40 MPa after 28 d. The nearly linear increase in strength over time reflects the proper progress of cement hydration, the formation of a dense microstructure, and the absence of deleterious side effects from additives. These samples serve as a benchmark for evaluating the impact of incorporating PU foam. This indicates that the mortar matured properly and without disturbances, and its structure remained compact and resistant to compressive loads. PU1 samples, containing 1% added foam, also exhibited a systematic increase in strength over time, reaching 38.4 MPa after 28 d. The difference from the reference samples was small, suggesting that a slight addition of foam reduces the strength but does not disqualify the material from structural applications. This level of modification may be acceptable, especially if the benefits of the foam (e.g., improved thermal insulation properties) are desirable. In the case of PU2 samples, containing 2% foam, a significant reduction in strength was observed, which, after 28 d, amounted to only 17.1 MPa. Despite maintaining an upward trend over time, the overall strength was much lower than that of PUO and PU1, and all specimens exhibited the expected trend of strength increase over time, confirming that PU foam does not chemically inhibit cement hydration. Rather, the reduction in mechanical performance is primarily a physical effect, stemming from increased void content, potential segregation of the foam, and weaker interfacial transition zones.

The largest reduction in strength was observed in PU3 samples, which contained 3% polyurethane foam. After only 2 days, the strength was just 4.3 MPa, and after 28 days, it reached only 6.7 MPa.

To quantify how much of the strength reduction observed in PU-modified mortars was caused by the increase in effective water content and how much resulted directly from the incorporation of PU waste, a comparative analysis was carried out based on the calculated effective w/c ratios and the compressive strength values at 28 days (Figure 7). For mixtures PU1–PU3, the effective w/c increased from 0.50 (PUO) to 0.556, 0.612, and 0.668, respectively. According to established empirical relationships (e.g., Abrams’ law), an increase in w/c from 0.50 to 0.56 would typically result in an approximate strength reduction of 10–12%, an increase to 0.61 in a reduction of around 18–22%, and an increase to 0.67 in a reduction of roughly 28–32%. These theoretical values were compared with the real reductions observed in this study:

- PU1: expected reduction due to w/c only ≈ 10–12%, while the measured reduction was 4% (40–38.4 MPa)
  → This indicates that PU at 1% does not weaken the matrix beyond what is expected from the increased water content, and may even partially compensate through micro-reinforcement.
- PU2: expected reduction due to w/c ≈ 18–22%, while the measured reduction was 57% (40–17.1 MPa).
  → Only ~35–40% of the reduction can be attributed to increased effective w/c, while the remaining ~60% is caused by the PU particles themselves (high porosity, weak ITZ, microdefects).
- PU3: expected reduction due to w/c ≈ 28–32%, while the measured reduction was 83% (40–6.7 MPa).
  → Increased w/c explains ≤ 1/3 of the reduction; ≥2/3 of the loss results directly from the PU addition.

This comparison confirms that the strength decrease in PU1 can be explained almost entirely by the increased water demand, while in PU2 and PU3, the governing factor is the structural effect of the PU modifier. The nonlinear trend from PU2 to PU3 further demonstrates that polyurethane waste acts not merely as a lightweight aggregate but also as a discontinuity-generating component that introduces additional porosity, reduces the effective load-bearing cross section, and weakens the interfacial transition zone. Thus, the sensitivity analysis clearly separates the contributions of effective water content and PU incorporation. While the workability-related increase in water content plays a role, particularly for PU1, the dominant mechanism governing strength reduction at higher PU levels is the material effect of the PU foam itself rather than changes in w/c. This distinction allows for a justified comparison between the mixtures and supports the interpretation of the mechanical and durability results presented in this study.

Despite these differences in strength, all samples exhibited a typical maturation process, with their strength increasing over time. This shows that the presence of foam alone does not prevent cement hydration, but it clearly influences the quality of the resulting structure. The analysis indicates that only a small amount of foam (up to 1%) should be considered in mortar production, particularly where additional physical properties, such as insulation, are important. Increasing the foam content beyond this level considerably deteriorates the mechanical properties of the material. The results shown in the graph align with findings in the scientific literature regarding the modification of concrete with lightweight additives, such as polyurethane (PU) foam. Numerous studies have demonstrated that adding porous or lightweight materials to cement mixes can enhance certain performance properties, like thermal insulation and reduced dead weight; however, this often results in a decline in mechanical properties, especially compressive strength. The authors of such studies include

- Jones and McCarthy (2005) [63] and Kearsley and Wainwright (2001) [64] highlight that additives with high porosity or closed-cell structures (such as foams) lead to loosening of the cement matrix. They generate micropores and voids that diminish the effective load-bearing surface of concrete, thereby reducing its strength [63,64].
- Ramamurthy et al. (2009) indicate that introducing lightweight additives (e.g., foams, fly ash, or ceramic beads) into concrete can significantly lower the overall weight, but exceeding 1–2% content can considerably weaken structural properties unless offset by additional modifications, like polymers or superplasticisers [65].
- Conversely, Amran et al. (2015) [66] found that when using PU foam, the uniformity of dispersion and pore size are vital; the more homogeneous the structure and the smaller the pores, the less strength decrease is observed. Their research confirms that with a low PU content (around 1%), a desirable balance between strength and thermal insulation enhancement can be achieved [66].

**Figure 9:**
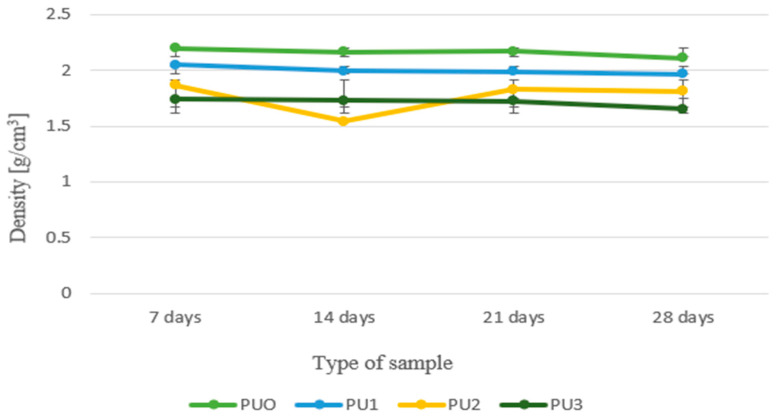
Change in mean density value when mortar samples are setting.

Analysing the results of the density of mortars modified with the addition of polyurethane (PU) foam across different maturation periods (7, 14, 21, and 28 d), a clear relationship between the foam content and the density of the obtained material can be observed (Figure 9).

The reference samples (PUO), which contained no additives, exhibited the highest and most stable density, approximately 2.2 g/cm^3^. This value is typical for traditional mortars with a compact structure and low porosity, confirming their complete, unmodified characteristics. Adding 1% foam (PU1) caused a slight decrease in density compared to the reference sample; this value fluctuated around 2.0 g/cm^3^ throughout the study period. The reduction is moderate and indicates that, at such a low level of addition, the material’s structure remains relatively dense, and the foam does not create significant air voids that destabilise the mortar. Larger changes were observed in the PU2 sample (2% foam), which experienced the greatest decrease in density on the 14th day of curing, reaching approximately 1.55 g/cm^3^. After this point, the density increased again and stabilised around 1.8 g/cm^3^. This behaviour may suggest ongoing structural changes in the material, possibly related to continued cement hydration and partial “healing” of the pores in the structure. The lowest density was observed in PU3 samples (3% foam), with values not exceeding 1.7 g/cm^3^. A consistent, clear trend was also evident: there was no significant increase in density over time, indicating that introducing 3% foam produces a permanent change in the mortar’s structure, associated with high porosity and low solid content per unit volume.

The conclusions of this analysis are clear: as the amount of PU foam in the concrete mix increases, the density of the material decreases significantly. This is desirable in the context of creating mortars that are characterised by lower dead weight; however, it should be remembered that the reduction in density is also associated with a deterioration in mechanical strength (as shown in the previous analysis). Therefore, a balance must be struck between the expected weight reduction and the level of the material’s structural properties.

The next phase of the evaluation involved analysing the behaviour of the tested cementitious mortars at varying negative temperatures (around −18 °C ± 2 °C) and positive temperatures (around +18 °C ± 2 °C), with simultaneous water exposure. After 25 cycles, the surface appearance, mass change, and mechanical properties of the samples were assessed. In all tested cases, no visible surface damage was observed with the naked eye during the evaluation of the samples. Table 5 shows the results of additional tests conducted after 25 cycles, specifically, changes in the weight of the samples. According to Polish standards established over the past 35 years and supported by extensive practical experience in the use of cement mortars in construction, samples after 25 freeze–thaw cycles should not exhibit cracks or damage, and their weight loss should not exceed 5%. Moreover, the reduction in compressive strength should be limited to 20% [61].

The results from the frost resistance test on mortar samples with added polyurethane (PU) foam revealed an unusual but consistent pattern of increased compressive strength after 25 freeze–thaw cycles across all samples, both reference and modified. This phenomenon can be explained by delayed cement hydration, supported by moisture availability and low temperatures, which supports previous findings by other researchers [61]. Such an effect is sometimes observed in concrete that has not fully matured before freezing; some hydration products may still form in the presence of moisture and low temperatures. Weight changes after 25 freeze–thaw cycles were small but differed depending on the sample type. The highest stability in terms of both strength and weight was observed in the PUO (reference) and PU1 (with 1% foam) samples. Their strength increased by + 9.2% and + 9.4%, respectively, with minimal weight variations. These results align with previous observations concerning density and strength. Both PUO and PU1 exhibited relatively high densities (over 2.2 g/cm^3^ for PUO and approximately 2.0 g/cm^3^ for PU 1) and higher compressive strength, indicating a compact and less absorbent structure. Therefore, it can be concluded that adding up to 1% polyurethane foam does not negatively impact resistance to cyclic conditions and may even enhance the concrete’s ability to further mature and stabilise its microstructure. Conversely, for PU 2 (2% PU) and PU3 (3% PU) samples, although there was an increase in strength after cyclic freeze–thaw, this was accompanied by more noticeable changes in weight; the weight gain of +1.6% in PU3 and the weight loss of −1.4% in PU2 are particularly concerning. This may suggest increased absorbency and susceptibility to water transport within the concrete structure modified with higher foam content. These findings are consistent with previous density analyses, where PU2 and PU3 samples displayed the lowest densities (below 1.8 g/cm^3^), indicating a more porous structure. Such porosity can facilitate water absorption and ion migration, potentially leading to reduced durability over time, especially with prolonged exposure to aggressive freezing conditions.

In the context of a previous study [67], similar trends were observed in foam concretes, where lower foaming additive content improved thermal insulation without significantly reducing strength, while higher foam percentages led to increased water absorption and a greater susceptibility to degradation. Therefore, it is crucial to optimise the amount of PU foam added, which will, on the one hand, reduce the concrete’s weight and, on the other, not negatively impact its resistance to weather conditions. PUO and PU2 samples exhibited a loss of mass, possibly indicating fine particle leaching or water migration from the structural pores. Conversely, PU1 and PU3 samples showed a slight increase in weight, likely due to water absorption into the porous foam structure, especially in PU3, where the foam addition is the highest and most absorbent. Although all samples demonstrated increased strength after the freeze–thaw cycles—suggesting a lack of structural deterioration—these results should be interpreted with caution.

- PUO and PU1 samples demonstrated very stable performance, with increased strength and minimal weight change, indicating good resistance to freeze–thaw cycles.
- The PU2 sample experienced the greatest weight loss (−1.4%), which may suggest a higher risk of degradation and a shorter service life under frost conditions.
- The PU3 sample, despite gaining strength, increased in weight (+1.6%), which could indicate high absorbency and a potential risk of future weakening of the material; water absorption during operation may lead to bursting during subsequent freezing cycles.

The results of this study show that adding polyurethane foam up to 1% by volume does not weaken the frost resistance of mortar and may even aid ongoing hydration and microstructural stability during freeze–thaw cycles. The increase in compressive strength after 25 freeze–thaw cycles, observed in both reference and modified samples, supports the idea of delayed cement hydration at low temperatures when moisture is present. On the other hand, higher foam contents (2–3%) were found to raise porosity, which led to larger mass changes after testing, indicating greater water absorption and a possible risk of long-term deterioration. These results align with previous studies [65,66,67] on foam concretes, where higher foaming levels caused increased water uptake and frost susceptibility. In summary, it can be concluded that the optimal polyurethane foam content should stay below approximately 1.5%, offering a good balance between reduced density, improved thermal insulation, and adequate frost durability.

To evaluate the reliability of the obtained mechanical performance results, a statistical assessment was carried out including standard deviation and coefficient of variation (CV). The analysis was performed separately for flexural strength, compressive strength, and compressive strength after the freeze–thaw test—Table 6.

Flexural strength—The coefficients of variation for mixtures PUO, PU1, and PU2 fall within the range of 7.7–8.4%, which is typical for cement mortars tested under controlled laboratory conditions. This indicates that these mixtures do not exhibit excessive variability, and the incorporation of 1% and 2% PU does not negatively affect measurement repeatability. A notable increase in the CV to 16.3% for PU3 demonstrates substantially reduced material homogeneity at 3% PU content, which is consistent with the visibly higher porosity of this mixture. These findings confirm that higher PU replacement levels lead to increased heterogeneity, which directly contributes to a larger spread of flexural strength values.

Compressive strength—The reference mortar (PUO) shows a very low coefficient of variation (1.1%), indicating highly stable and consistent strength measurements. In PU1 and PU2, the CV increases to 2.8% and 7.2%, respectively, though these values remain acceptable for modified mortars with increased porosity. The highest variability is observed for PU3 (16.6%), demonstrating clear deterioration in homogeneity and a greater sensitivity of the material to local pore distribution as PU content increases. This statistical pattern supports the observed trend of reduced compressive strength at higher PU levels.

Compressive strength after 25 freeze–thaw cycles—After 25 freeze–thaw cycles, the CV values for PUO and PU1 remain low (2.0–2.1%), suggesting that the material structure of these mixtures remains relatively stable even under cyclic thermal loading. For PU2 and PU3, the CV increases to 6.0% and 13.3%, respectively, but these values are still slightly lower than those recorded prior to freeze–thaw exposure. This may indicate partial microstructural refinement or additional hydration during the freeze–thaw regime. Nonetheless, the elevated CV in PU3 again reflects its higher porosity and reduced structural uniformity.

Based on the research, it was found that

- Mixtures PUO–PU2 exhibit acceptable variability, confirming good measurement reliability and stable material behaviour.
- PU3 consistently shows the highest variability, both before and after freeze–thaw cycles, indicating significant heterogeneity linked to excessive porosity at 3% PU replacement.
- A low PU dosage (1%) maintains excellent stability of results, with variability comparable to the reference mixture.
- The statistical descriptors demonstrate that the observed strength reductions at 2–3% PU and the partial strength increases after freeze–thaw cycles are not random effects but reflect actual material-related changes in microstructure and porosity.

Water absorption tests demonstrated a clear link between the amount of polyurethane (PU) waste and the absorbency of cement mortars. As the PU content increased, so did the absorbability consistently. The reference sample showed an absorbency of 8.8%. Adding 1% PU (PU1) raised water absorption to 11.6%, an increase of 2.8 percentage points compared to the sample without the additive. For PU2 mortar (2% PU), absorbability reached 15.8%, and for PU3 (3% PU), it was 21.9%. Thus, higher PU levels led to a consistent and significant rise in the mortars’ water absorption capacity, as shown in Table 7. The greatest increase in water absorption, by 13.1 percentage points compared to PUO, was observed in mortar containing 3% PU. It should also be noted that the absorption value obtained for PU3 (21.9%) exceeds the typical thresholds defined in EN standards for masonry mortars intended for unprotected exterior exposure (<10–14%), which may limit its applicability in direct outdoor conditions. Polyurethane waste likely possesses a cellular structure (open- or semi-closed cell), which promotes water absorption. When incorporated into mortar, PU acts as a micro reservoir of moisture, increasing the available space for water. To achieve comparable consistency, the PU-infused mortars required additional water. A higher water/cement ratio (w/c) influences the increase in structural porosity after setting and promotes the formation of micro voids that facilitate water absorption.

A decrease in the density of the PU2 and PU3 mortars (to below 1.7 g/cm^3^) indicates a higher presence of air voids and lower compactness of the structure, which leads to increased capillary absorbability. Greater absorbency may cause reduced frost resistance, especially in cyclically wet and frozen environments.

### 3.3. Microstructure

**Figure 10:**
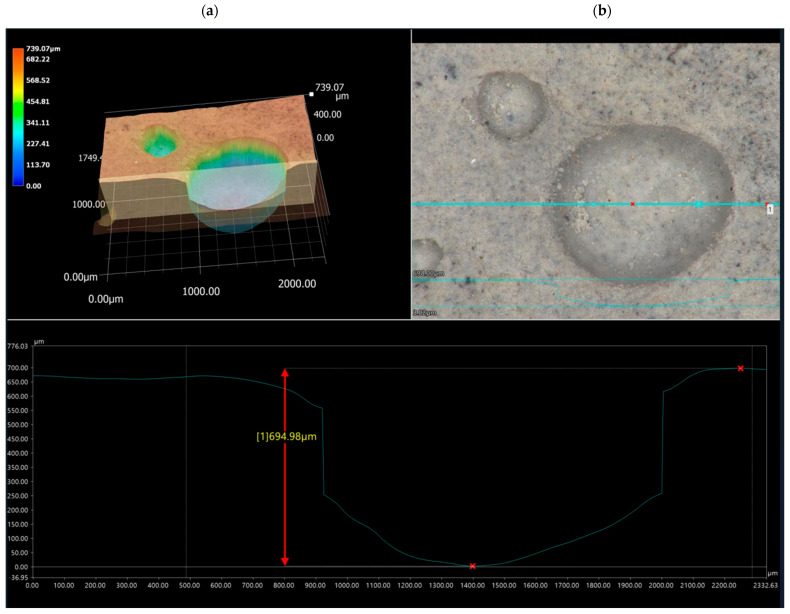
(**a**) Surface of the reference sample without the addition of polyurethane foam. (**b**) The photo shows a 3D image of the selected fragment of the sample surface, 30× magnification of the selected unevenness, and measurement of surface irregularities.

**Figure 11:**
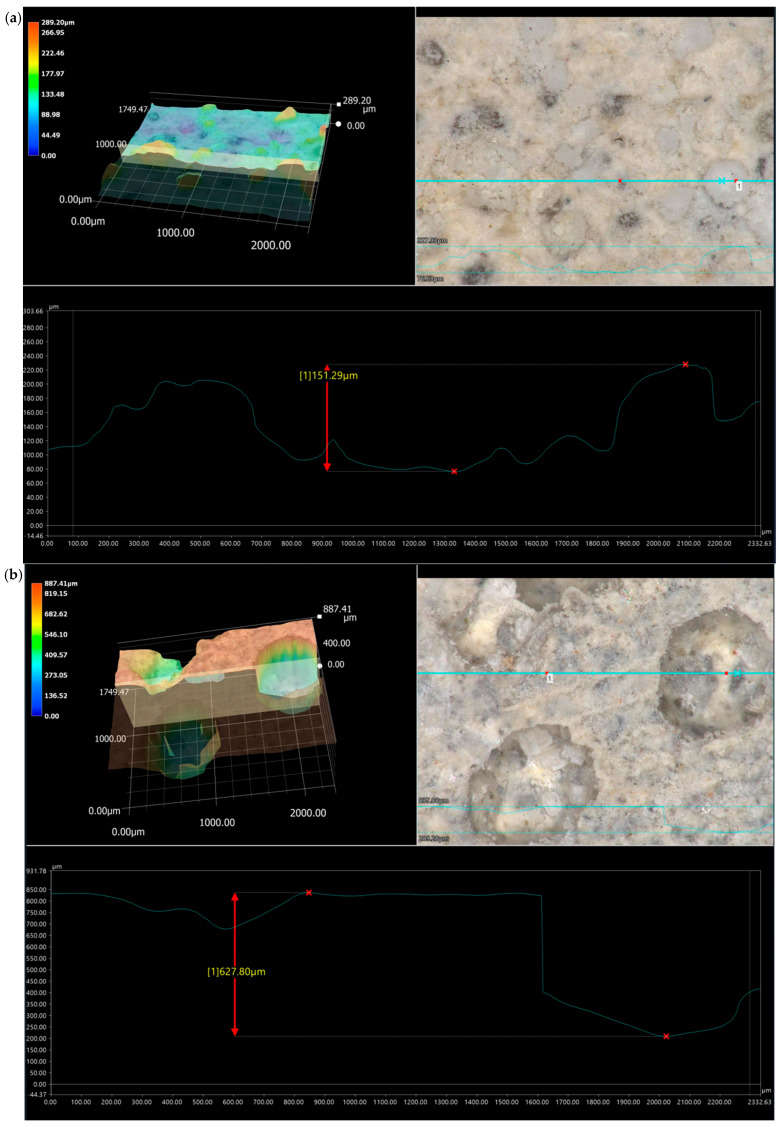
Specimen surface with 1% polyurethane foam added. (**a**) Reference sample; (**b**) sample after 25 freeze–thaw cycles in the presence of water.

**Figure 12:**
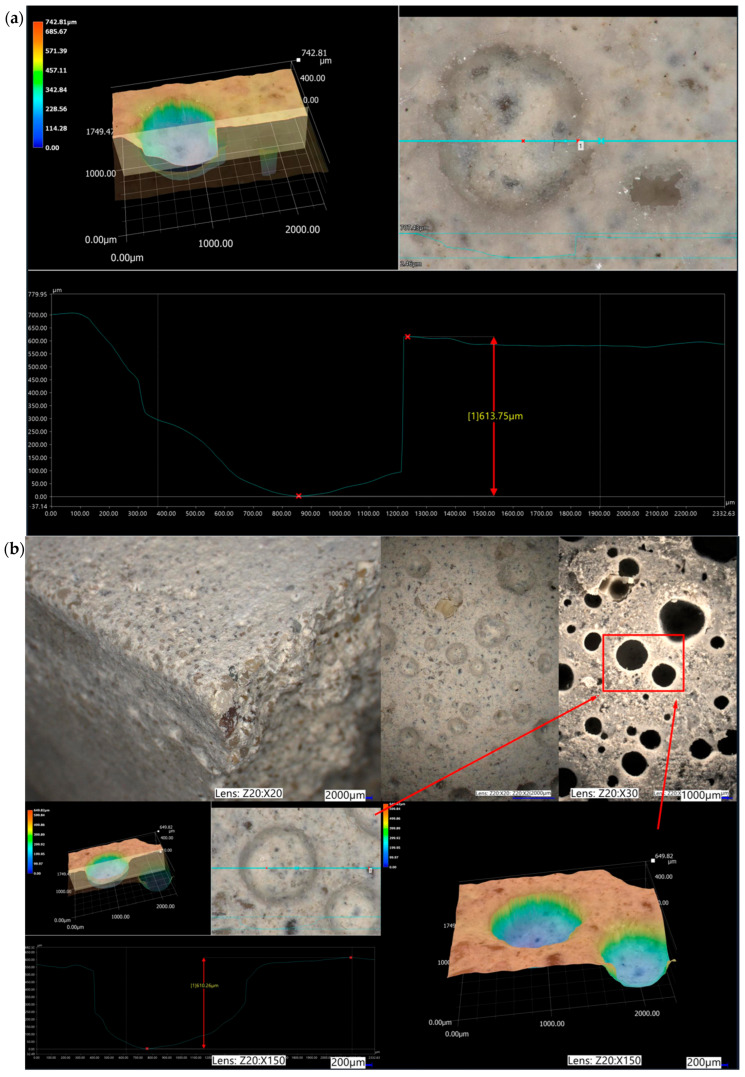
Specimen surface with 2% polyurethane foam added: (**a**) reference specimen; (**b**) specimen after 25 freeze–thaw cycles in the presence of water.

**Figure 13:**
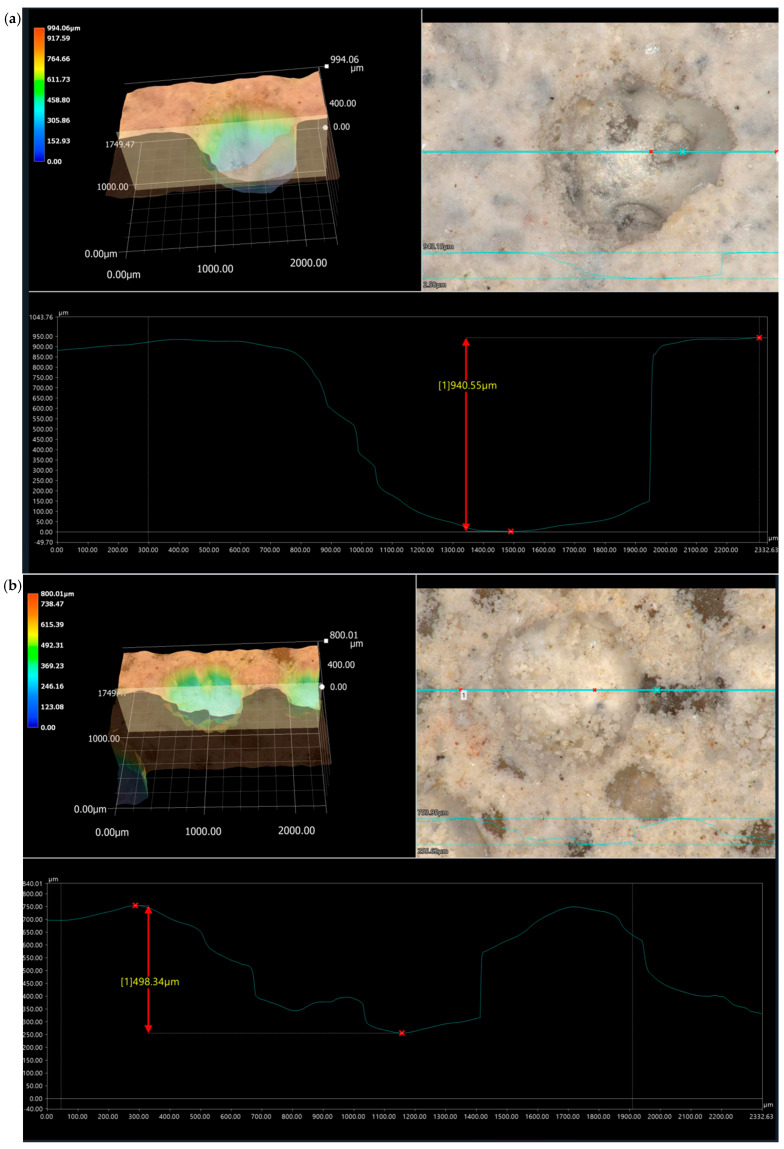
Sample surface with 3% polyurethane foam added: (**a**) reference sample and (**b**) sample after 25 freeze/thaw cycles in the presence of water.

The assessment of the surface microstructure reveals that the presence of dusty polyurethane waste does not significantly impact surface evenness, both in specimens not subjected to ageing loads and after varying freeze–thaw cycles in water. Across all examined surfaces, including mixtures with 0%, 1%, 2%, and 3% PU, maximum surface irregularities ranged from approximately 150 μm to 950 μm, with notable peaks near 400 μm and 600 μm, as shown in Figure 10, Figure 11, Figure 12 and Figure 13. These irregularities were not caused by the loss of the modifier but rather by sand fractions or voids formed after water droplets evaporated. Visible surface damage was not detectable with the “naked eye” in any of the evaluated cases. No polyurethane foam grains appeared on the surface of the tested samples, indicating that during the setting of the cement mortars, it did not float to the surface.

The photos show 3D images of the examined surfaces, 30× magnification of the selected surface fragment, and the value of the average profile unevenness.

**Figure 14:**
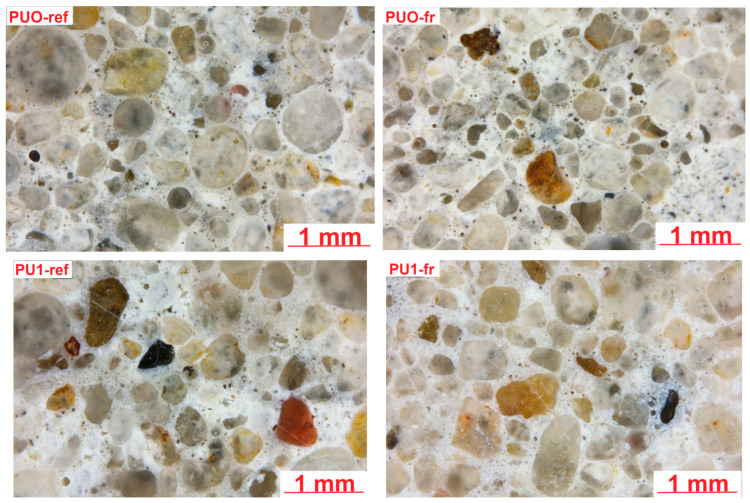
Microstructure of examined samples (optical microscopy).

Microscopic analysis was conducted to evaluate the quality of bonding between the PU particles and the cement matrix. Furthermore, the influence of repeated freeze–thaw cycles on the microstructure of the reference mortar (PUO-fr) and the mortar containing polyurethane (PU1-fr) was systematically examined in order to assess the potential impact of PU incorporation on the long-term durability and frost resistance of the cement composite. Figure 14 presents an example of the microstructure of the reference mortars, PUO and PU1, examined using optical microscopy.

Microstructural analysis via optical microscopy identified several macropores, primarily spherical. The grains of the fine aggregates mainly consisted of quartz. No cracks were observed in the microstructure, even in samples subjected to freeze–thaw cycles. Further investigations were conducted using SEM.

**Figure 15:**
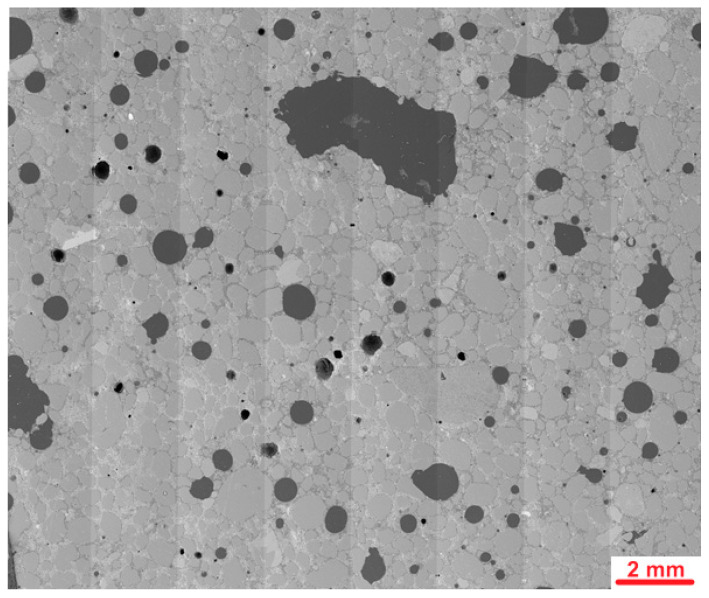
Stitched SEM images of the PUO reference sample.

Figure 15 presents the stitched SEM images of the investigated area of the PUO reference sample, which was not subjected to freeze–thaw cycles.

**Figure 16:**
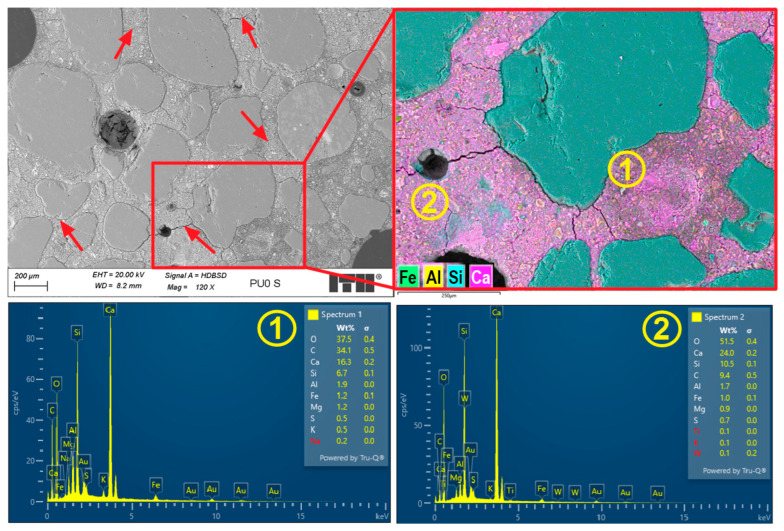
Microstructure of PUO-ref sample. Red arrows mark cracks; areas 1 and 2 show different morphology and composition of the C-S-H phase.

The microstructure of the PUO reference sample showed several macropores, most of which were spherical. Some of them were larger and had irregular shapes. Figure 16 presents an example of the microstructure of the PUO-ref sample during deeper investigations.

Analysis of the microstructure of the PUO-ref sample, which was not affected by the freeze/thaw cycles, revealed some cracks in the cement matrix. Some of the observed cracks radiate from the aggregate grains or air voids, suggesting that they may form during the shrinkage process in the early mortar. In the cement matrix, regions of the C-S-H phase with varying compositions and morphologies were identified, as shown in Figure 16, where area 2 had a higher concentration of calcium and silicon than area 1. Area 1 exhibited greater porosity than area 2. This difference could be due to a higher carbon signal detected in area 1, likely from the resin, which appears to have partially filled the micropores. These observations may result from the uneven mixing process, which caused differing water–cement ratios across various areas.

**Figure 17:**
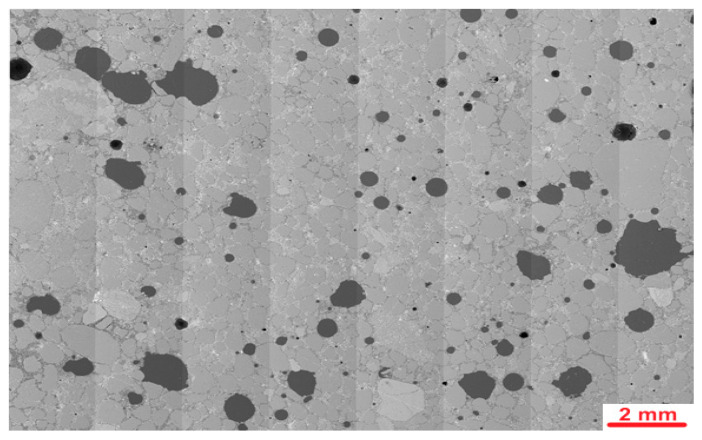
Stitched SEM images of PUO-fr sample.

Figure 17 presents the stitched SEM images of the PUO-fr sample after the freeze–thaw cycles.

**Figure 18:**
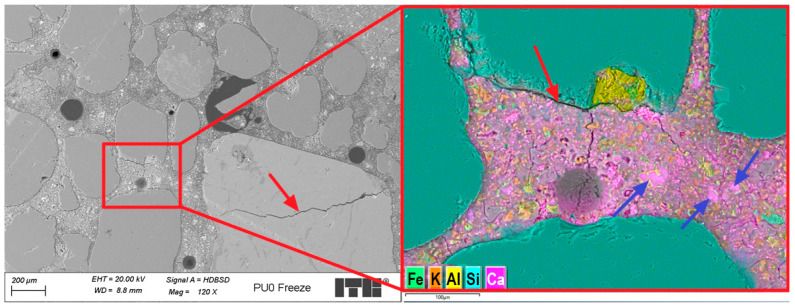
Microstructure of PUO-fr sample. Red arrows mark cracks; blue arrows mark relicts of cement clinker.

The analysis of the PUO-fr sample showed great similarity to that observed in the PUO-ref sample. Several macropores were also observed, most of which had a spherical shape, excluding the larger one with an irregular form. Figure 18 presents an example of an image collected during the deeper examinations of the PUO-fr sample.

Investigations of the microstructure of sample PUO-fr after freeze–thaw cycles revealed several cracks, mainly in the grains of the aggregate, which were not present in the reference sample. This suggests that some of the aggregate grains were not frost-resistant. The cement grout also cracked, similar to the reference sample, but the pattern of cracking was different. In the sample after freeze–thaw cycles, the cracks resembled those caused by frost action rather than shrinkage. Typical shrinkage cracks were not observed in the PUO-ref sample, and the by-products of sealing these cracks with portlandite were also not observed.

**Figure 19:**
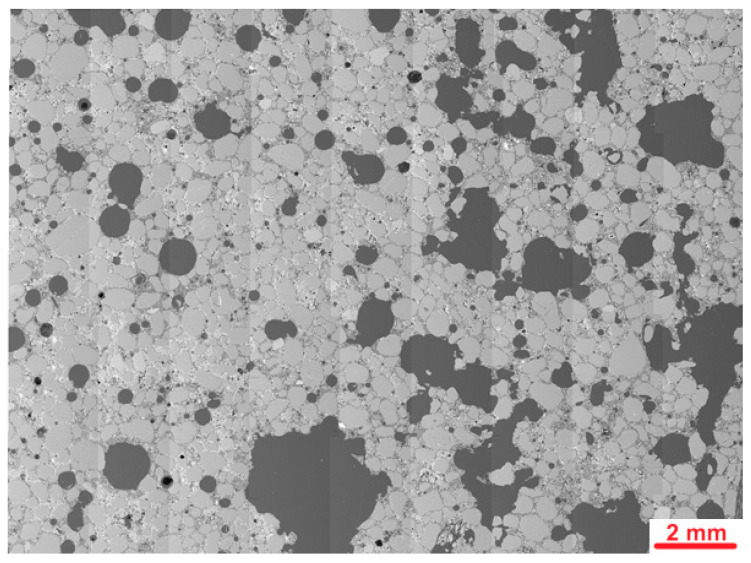
Stitched SEM images of PU1.

Figure 19 presents the stitched SEM images of the PU1 sample.

**Figure 20:**
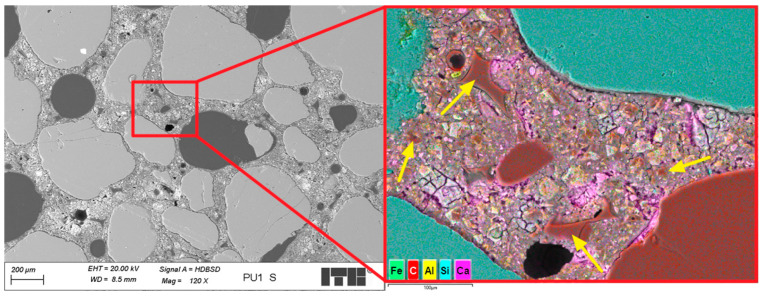
Microstructure of PU1 sample; arrows mark PU foam grains.

Investigations of the microstructure of PU1 mortar containing the addition of PU showed many macropores, from which the majority were irregular in shape. The amount of air voids was greater than that observed in the PUO sample without the addition of PU foam. The shape and size of pores probably do not increase the frost resistance of mortars. Higher porosity might be caused by the air entering ability of PU foam or by decreased workability and harder compaction of samples during moulding. Figure 20 presents an example of the images collected during further microstructural observations.

**Figure 21:**
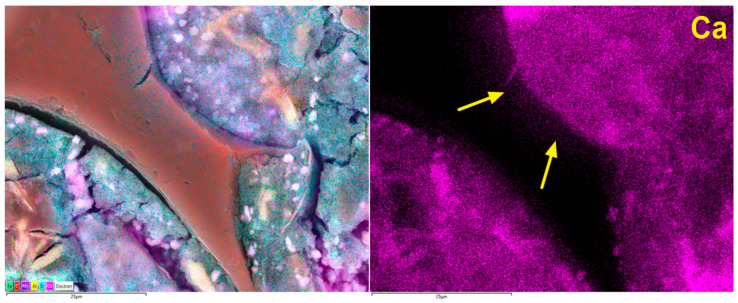
Microstructure of PU1 sample in the area with PU foam grain; arrows mark migration of calcium ions.

The analysis of the microstructure of the PU1 mortar sample containing PU foam revealed macro- and micropores and grains of fine quartz aggregates. Clinker relicts were observed in the cement grout. PU foam in the cement matrix was well surrounded by the C-S-H phase; however, the transition zone was sometimes sealed and tight, whereas in other cases, it was discontinuous and cracked. About 70% of interfaces showed tight bonding, while 30% exhibited microcracking. In some areas near the PU foam grains, the porosity and composition of the C-S-H phase were modified, probably due to the influence of PU foam grains on the local water–cement ratio. Figure 21 shows a closer look at the transition zone between the PU foam and cement grout.

Analysis of the transition zone between the cement grout and PU foam grains revealed that its characteristics can vary. This variation depends on the water content in the PU foam grains and their porosity. When the water content is low, the transition zone is sealed and compact, with relatively good adhesion between the cement grout and foam grains. The migration of Ca ions was observed (Figure 21) due to the entry of calcium hydroxide solution into the microstructure of the foam. This effect also locally decreases the water–cement ratio, which may enhance the compressive strength of the composite. However, other foam grains with lower open porosity or higher initial water content do not exhibit such an ability to draw calcium hydroxide solution further into their microstructure, as no Ca ion migration was observed, and the modification of the C-S-H phase composition was neglected. During the microstructural analysis of the PU1 sample, it was difficult to determine which effect had a greater influence on the macroscopic properties of the composite. Nonetheless, the initial water content in the PU foam before mixing it with the fresh mortar was crucial not only for workability but also for the subsequent properties of the hardened cement composite.

**Figure 22:**
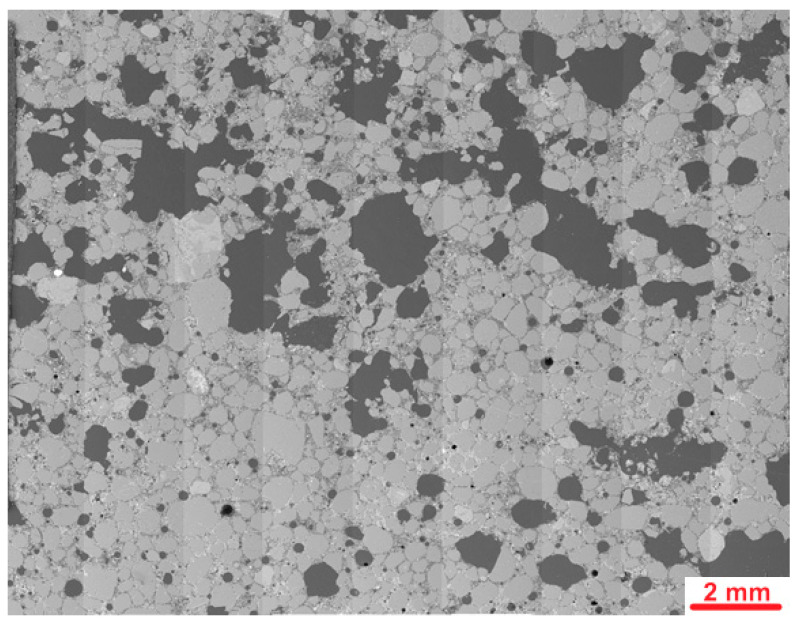
Stitched SEM images of PU1-fr sample.

Figure 22 presents the stitched SEM images of the PU1-fr sample after the freeze–thaw cycles.

**Figure 23:**
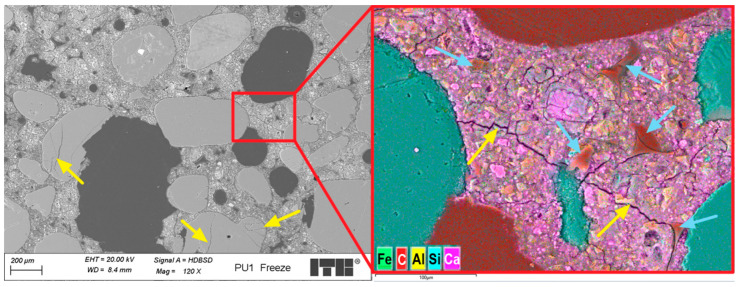
Microstructure of PU1-fr sample. Yellow arrows mark microcracks; blue arrows mark PU foam grains.

Observations of the microstructure of the PU1-fr sample after freeze–thaw cycles revealed several macropores like those observed in the PU1-ref sample, and macrocracks were not visible at the first spot as the typical damage caused by frost aggression. Figure 23 presents an example of the images collected during further, deeper investigations into the microstructure of the PU1-fr sample.

A detailed analysis of the microstructure of the PU1-fr sample after freeze–thaw cycles revealed several microcracks, likely caused by frost damage. Some cracks were within the quartz aggregate grains, while others extended through the cement matrix and the transition zone between the aggregates and cement grout. Cracks near the PU foam grains were also observed, but it remains unclear whether these grains prevent further crack propagation or initiate cracking. If air voids replace PU foam grains, they might reduce capillary water absorption and prevent damage from freezing water. However, since PU foam is porous, it can absorb water, and during freezing, this water expands, leading to cracking. If PU foam grains encapsulate air within the micropores, they could act as a mechanical buffer, reducing internal stress and preventing damage. An important consideration regarding the use of PU foam additives is their pore size distribution. This property was tested using statistical image analysis. Table 8 presents results of performed analysis.

Analysis of pore size distribution in mortars before and after freeze/thaw cycles showed that in PUO samples, frost aggression significantly changed the porosity parameters. Total porosity increased by about 30%. The number of pores increased about four times. Also, the maximum pore size doubled. However, average and median values decreased, which might have been caused by the formation of several micropores. The observed changes in pore size distribution in PUO samples occurred in the microstructure during freeze/thaw cycles. Analysis of changes in PU1 samples caused by frost action showed differences compared to PUO samples. Freeze/thaw cycles did not have much of an effect on total porosity, which increased by about 6%. However initial total porosity was several times higher than in PUO. Total porosity increased mostly due to a higher number of irregular pores, which do not prevent frost action. The number of pores in the PU1 sample after freeze cycles slightly decreased, while median and average values increased, which might have been caused by merging pores during degradation of the microstructure. Pore size distribution analyses have shown that similar strength losses in the PUO and PU1 samples after freeze/thaw cycles are due to different mechanisms of changes in the microstructure of the mortars.

## 4. Conclusions

This study presents an analysis of the durability of cementitious mortars with the addition of polyurethane foam (in amounts of 1, 2, and 3% by sand content) under a range of temperature exposures, including both cyclic positive and negative temperatures from −18 ± 2 °C to +18 ± 2 °C, as well as extreme positive temperatures. Given the limited number of mixture proportions tested and the small sample size, the results cannot be generalised, but should be regarded as significant observations that warrant further investigation. Based on these tests, the following conclusions can be drawn:

- Tests of mortars with the addition of polyurethane foam waste confirmed expectations regarding the use of lightweight materials, such as a reduction in density (from 9% for 1% foam additive to 23% for a 3% additive). However, this was accompanied by a simultaneous decrease in flexural strength compared to the reference sample, ranging from 15% to 70%, respectively. This was due to the increased porosity of the cement matrix. Because excessive foam (2–3%) causes significant deterioration of mechanical properties, it limits the potential for using such mixtures in structures exposed to mechanical loads and environmental conditions.
- All samples (PU 1, PU 2, PU3) showed an increase in compressive strength after 25 freeze–thaw cycles (of 9.0%), suggesting a beneficial effect of secondary cement hydration under cyclic freezing conditions. Simultaneously, the weight changes were minimal (from 0.1% to 1.6%), demonstrating the good structural resistance of the materials.
- The optimal content of polyurethane foam appears to be 1% of the sand mass (PU1); at this concentration, the most favourable compromise between strength, reduced density, and frost resistance was achieved. In this case, density decreased by 9%, and the reduction in bending strength was 15%, while the compressive strength decreased slightly, by 4%. Water absorption increased by 2.8% compared to the sample without the additive. After freeze–thaw cycles, it was found that the compressive strength increased by approximately 9.5%, with a corresponding weight change of 0.1%.
- The absorbability of cement mortars increased almost proportionally to the growth of PU content, by 2.8%, 15.8%, and 21.9%. The primary reason for this is a combination of the waste material’s porosity and the increased amount of water required to maintain consistency.
- The addition of the dust fraction of polyurethane foam to cement mortars in the amounts of 1%, 2%, and 3% of the aggregate weight does not affect the change in the surface evenness of the samples, both unaged and after 25 freeze–thaw cycles.
- The analysis of the microstructure of mortars showed that the addition of PU foam grains to mortar increased the content of macropores, which might be caused by the decreased workability due to the high-water demand of the additive. The grains of PU foam might play the role of an internal water buffer during the early stage of curing the cement composite, which might be beneficial. The most important issue regarding the addition of PU foam to the cement composite is the initial water content in the PU foam before adding it to the cement mix, as a higher water content will help avoid problems with workability but will locally increase the water–cement ratio in the cement matrix and may weaken the transition zone. A lower initial water content in the PU foam caused the opposite effect. The role of PU foam in preventing frost aggression appears to depend strongly on the pore size distribution and its characteristics (open/closed) in the foam. Open pores may increase water absorption and maintain water during freezing, while closed pores with entrapped air may play a role in tension buffering and prevent microdamage caused by frost action. Analysis of the pore size distribution showed that the mechanisms of change in the microstructure of PUO and PU1 mortars exposed to freeze/thaw cycles are different, despite a similar effect on compressive strength.

The findings show that adding lightweight, porous components, such as polyurethane foam, can be effectively incorporated into cementitious mortar technology; however, careful optimisation of the mixture composition is necessary. Therefore, in construction applications, it is crucial to balance the PU additive content suitably. A small amount, up to 1%, may be acceptable, particularly as it can enhance other material parameters such as thermal insulation or porosity. Nonetheless, for applications exposed to extreme temperatures above zero, it is advisable to limit the PU content or incorporate additional flame retardants.

## Figures and Tables

**Table 1:** Physical properties and phase composition of CEM I 42.5 R cement (data from the manufacturer) [52].

Specific Surface Area According to Blaine [cm^2^/g]	Start of Setting Time [min]	Compressive Strength After 2 d [MPa]	Compressive Strength After 28 d [MPa]
3332	219	21.1	49.7
Proportion of CEM I mineral phases [%]			
C_3_S–55.64 Tricalcium silicate (alite)	C_2_S–14.57 Dicalcium silicate (belite)	C_3_A–8.17 Tricalcium aluminate	C_4_AF–6.82 Tetracalcium aluminoferrite

**Table 2:** Chemical properties of CEM I 42.5R cement (data from the manufacturer) [52].

Loss of Ignition [%]	SO_3_ Sulphate Content [%]	Cl Chloride Content [%]	Alkali Content as Na_2_O_eq_ [%]	SiO_2_ [%]
3.21	2.97	0.05	0.77	20.21
Al_2_O_3_	Fe_2_O_3_	CaO	CaOw	MgO
4.40	2.43	64.36	1.99	1.98

**Table 3:** Properties of polyurethane foam of a full-value product from which the waste used in the research was produced (data from the manufacturer) [55].

Properties	Mean	Properties of Polyurethane Foam
Width [cm]	130.2	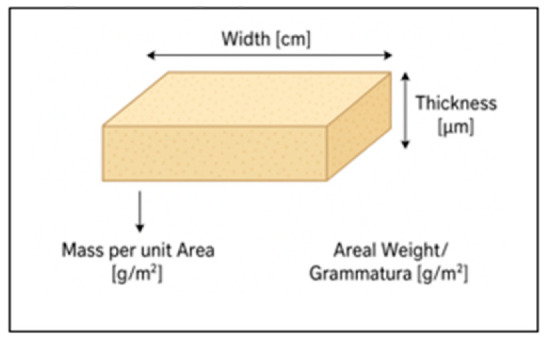
Mass per unit Area [g/m^2^]	189.4	
Warp density [threads/10 cm]	85.2	
Weft density [threads/10 cm]	83.0	
Areal Weight/Grammatura [g/m^2^]	125 ± 4%	
Thickness/spessore [µm]	170 ± 15%	

**Table 4:** Composition of cementitious mortars.

Weight of Ingredients [g]						
Test Sets’ Number	Aggregate	Water	Cement	PU Waste	Absorbed Water	Effective Water
PUO	1350.0	225.0	450.0	-	-	-
PU1	1336.5	255.0	450.0	13.5	4.7	250.3
PU2	1323.0	285.0	450.0	27.0	9.5	275.5
PU3	1309.5	315.0	450.0	40.5	14.2	300.8

**Table 5:** Average decrease in strength and average loss in mass of samples subjected to 25 cycles of freezing–thawing (+ indicates increase;–indicates decrease).

Sample Type	Compressive Strength, Average Value [MPa]		Average Increase/Decrease in Strength of Samples Subjected to 25 Freeze/Thaw Cycles [%]	Weight of Samples Subjected to 25 Freeze/Thaw Cycles, Mean Value [g]		Mean Weight Loss [%]
	Reference Samples	After 25 Freezing Cycles/ Defrosting		Before Ageing Cycles	After Ageing Cycles	
PUO	46.9	51.2	+9.2	565.2	562.0	−0.6
PU1	38.4	42.0	+9.4	521.6	521.7	+0.1
PU2	17.1	18.6	+8.8	483.5	476.9	−1.4
PU3	6.7	7.6	+13.4	444.5	451.8	+1.6

**Table 8:** Pore size analysis.

Property	PUOs	PU0-fr	PU1-s	PU1-fr
Total porosity [%]	8.5	11.4	23.5	24.9
Number of analysed pores	571	2312	5126	4640
**Pore Diameter [μm]**				
Minimum	3	3	3	3
Maximum	1599	3308	3313	3017
Average	81	35	30	33
Median	15	9	9	10

**Table 6:** Statistical analysis of strength test results for PU-modified cement mortars.

Specification	Flexural Strength			
	PUO	PU1	PU2	PU3
Standard deviation	0.264	0.244	0.264	0.164
Index of variation [%]	7.7	8.4	7.7	16.3
	**Compressive Strength**			
Standard deviation	0.447	1.066	1.235	1.111
Index of variation [%]	1.1	2.8	7.2	16.6
	**Compressive Strength After Frost Resistance Test**			
Standard deviation	1.037	0.867	1.110	1.017
Index of variation [%]	2.0	2.1	6.0	13.3

**Table 7:** Results of water absorption of cementitious mortars.

Sample Type	Water Absorption [%]	Change from PUO	Relative Change [%]
PUO	8.8	-	-
PU1	11.6	+2.8	+31.8
PU2	15.8	+7.0	+79.5
PU3	21.9	+13.1	+148.9

## Data Availability

The original contributions presented in this study are included in the article. Further inquiries can be directed to the corresponding author.
